# Is Vasoactive-Inotropic Score a Predictor for Mortality and Morbidity in Patients Undergoing Coronary Artery Bypass Surgery?

**DOI:** 10.21470/1678-9741-2020-0219

**Published:** 2021

**Authors:** Pınar Karaca Baysal, Füsun Güzelmeriç, Ersin Kahraman, Mustafa Emre Gürcü, Atakan Erkılınç, Tülay Orki

**Affiliations:** 1 Department of Anesthesiology and Reanimation, Kartal Koşuyolu Yüksek İhtisas Training and Research Hospital, Istanbul, Turkey.

**Keywords:** Length of Stay, Cardiopulmonary Bypass, Stroke Volume, Coronary Artery Bypass, Heart-Lung Machine, Morbidity, Intensive Care Units.

## Abstract

**Introduction:**

We aimed to investigate whether vasoactive-inotropic score (VIS) is a predictor for early postoperative morbidity and mortality.

**Methods:**

This study was planned as a prospective cohort study, between Nov 20 2018 and May 15 2019, including a total of 290 patients aged 20 years or older who underwent elective on-pump coronary artery bypass grafting (CABG). Patients’ demographic data, aortic cross-clamp and cardiopulmonary bypass times, European System for Cardiac Operative Risk Evaluation (EuroSCORE) score, cardiac ejection fraction (EF), VIS, intubation duration, and intensive care unit length of stay were recorded. Postoperative mortality and morbidity were recorded. Hourly doses of inotropes for VIS were recorded for each patient, and VIS was calculated.

**Results:**

Among the cases, 222 (77%) were male and 68 (23%) were female. The mean age of our patients was 62.5 years (37-86). Combined morbidity and mortality rates of our patients were 23.8%. An optimal cutoff point for VIS of 5.5 could predict combined morbidity and mortality with 90% sensitivity and 88% specificity. Low EF, prolonged operation time, high EuroSCORE, and high VIS are independent factors in the early postoperative period for the development of combined morbidity and mortality in patients who underwent elective CABG.

**Conclusion:**

VIS is the most critical and EuroSCORE is the second most important scoring systems. They independently predict combined morbidity and mortality in undergoing elective coronary artery bypass surgery.

**Table:** 

Abbreviations, acronyms & symbols	
ACC	= Aortic cross-clamp
AuROC	= Area under the receiver operating characteristics
CABG	= Coronary artery bypass grafting
CI	= Confidence interval
CPB	= Cardiopulmonary bypass
EF	= Ejection fraction
EuroSCORE	= European System for Cardiac Operative Risk Evaluation
ICU	= Intensive care unit
IS	= Inotropic score
OR	= Odds ratio
PDE	= Phosphodiesterase inhibitor
ROC	= Receiver operating characteristics
SE	= Standard error
VIS	= Vasoactive-inotropic score

## INTRODUCTION

Compared to previous years, the mortality rate in open-heart surgery has decreased with the development of surgical technique and medical treatment^[1]^. In contrast, the morbidity rate continues to rise with the increasing number of elderly and high-risk patients^[1]^. Cardiac dysfunction and intraoperative factors are the most important predictors for mortality and morbidity in open-heart surgery^[2-5]^. Although there are some parameters that can predict this situation, there is not a specific predictive scoring system calculated with intraoperative parameters yet^[6]^.

Vasoactive treatment is generally performed during surgery by taking into consideration the patients' clinical parameters^[7]^. A simple numerical formula that calculates the vasoactive-inotropic score (VIS) was firstly used in infants who underwent an arterial switch operation^[7]^. VIS has been used to determine the postoperative morbidity and mortality risk of adult patients who had open-heart surgery^[6]^.

We aimed to investigate whether VIS is a predictor for early postoperative morbidity and mortality.

## METHODS

This prospective study was conducted between Nov 20 2018 and May 15 2019 upon the approval of our institution's ethics committee (2018/6/69), and the data were evaluated retrospectively. Patients aged 20 years or older who underwent elective on-pump coronary artery bypass grafting (CABG) were included in the study. Different surgical teams performed CABG on the patients, but the anesthesia team (PKB, AE, MEG) was the same.

Patients with ventricular assist devices or intra-aortic balloon pumps, valvar surgeries, emergency cardiac operations, cardiac revision operations, and pediatric patients were excluded from the study.

Demographic data, aortic cross-clamp (ACC) and cardiopulmonary bypass (CPB) times, European System for Cardiac Operative Risk Evaluation (EuroSCORE) score, ejection fraction (EF), VIS, intubation duration, intensive care unit (ICU) length of stay, postoperative mortality, and morbidity were recorded.

Mechanic circulatory support, renal insufficiency, cardiac arrest, cardiac arrhythmia, and central nervous system damage were the cause of morbidity and mortality in the postoperative period.

Renal insufficiency was determined according to the RIFLE classification (*R* for risk, *I* for injury, *F* for failure, *L* for loss of kidney function, and *E* for end-stage renal disease)^[8,9]^.

Patients who developed morbidity and mortality or not were divided into two groups. The patients’ characteristics and operation data were compared in Table 1.

**Table 1:** Clinical characteristics of research groups.

	Total	Morbidity and mortality group (n=69)	Good outcome group (n=221)	*P*-value*
Age	62.5 (37-86)	64 (39-86)	61.5 (37-78)	0.03
Sex (male/female)	68 (23%)/222 (77%)	19 (27.5 %)/50 (72.5%)	50 (22.6%)/171 (77.4 %)	0.2
Average EuroSCORE	0.98 (0.5-6.75)	1.99 (9.55-6.75)	0.82 (0.51-4.98)	0.001
EF	55 (30-65)	50 (30-60)	65 (35-65)	0.001
< 50%	202 (69.7%)	29 (42%)	173 (78.3%)	
> 50%	88 (30.3%)	40 (58%)	48 (21.7%)	
Operation data				
CPB time (min)	115.5 (38-260)	146 (82-260)	105 (38-201)	0.001
ACC time (min)	68 (18-200)	78 (43-200)	63 (30-65)	0.001
Intubation duration (hours)	7 (1.7-89.5)	13.25 (10.83-89.5)	5.5 (1.7-16.33)	0.02
ICU length of stay (hours)	47 (12.7-720)	132.5 (72-720)	44.7 (12.7-150)	0.03
VIS < 5.5	218 (75.2%)	15 (21.7%)	203 (91.8%)	0.001
VIS > 5.5	72 (24.8%)	54 (78.2%)	18 (8.1%)	0.003

ACC=aortic cross-clamp; CPB=cardiopulmonary bypass; EF=ejection fraction; EuroSCORE=European System for Cardiac Operative Risk Evaluation; ICU=intensive care unit; VIS=vasoactive-inotropic score

* The *P*-value is for the Chi-square test for categorical variables and Mann–Whitney U test for continuous variables for between-group comparisons. Data are presented as n (%) for categorical variables and median (25^th^–75^th^ percentiles) for continuous variables

Inotropic drug doses were recorded hourly for each patient. VIS was calculated as dopamine dose (µg kg^-1^ min^-1^) + dobutamine dose (µg kg^-1^ min^-1^) + 100 × epinephrine dose (µg kg^-1^ min^-1^) + 100 × norepinephrine dose (µg kg^-1^ min^-1^) + 10,000 × vasopressin dose (U kg^-1^ min^-1^) + 10 × milrinone dose (µg kg^-1^ min^-1^)^[7]^.VIS value was calculated once when the patient's clinical data were most stable, at the end of the operation, and before the patient was transferred to the ICU.

### Statistical Analysis

Data analysis was carried out by using the IBM Corp. Released 2013, IBM SPSS Statistics for Windows, Version 22.0, Armonk, NY: IBM Corp. software. Results were presented as mean ± standard deviation or median for continuous variables and numbers (percentage) for nominal/categorical variables. Comparison between groups was performed by Chi-square test, Mann-Whitney U test, or t-test, as appropriate. The diagnostic accuracy of VIS was analyzed by calculating the area under the receiver operating characteristics (AUROC) curve. We performed a binary logistic regression analysis to find independent factors causing morbidity and mortality. We calculated sensitivity, specificity, and negative and positive predictive values, with 95% confidence interval (CI). A P-value < 0.05 was considered statistically significant.

## RESULTS

Among the 290 patients who underwent elective CABG included in the study, 77% (222/290) were males and 23% (68/290) were females. The mean age of our patients was 62.5 years (37-86).

**Fig. 1:**
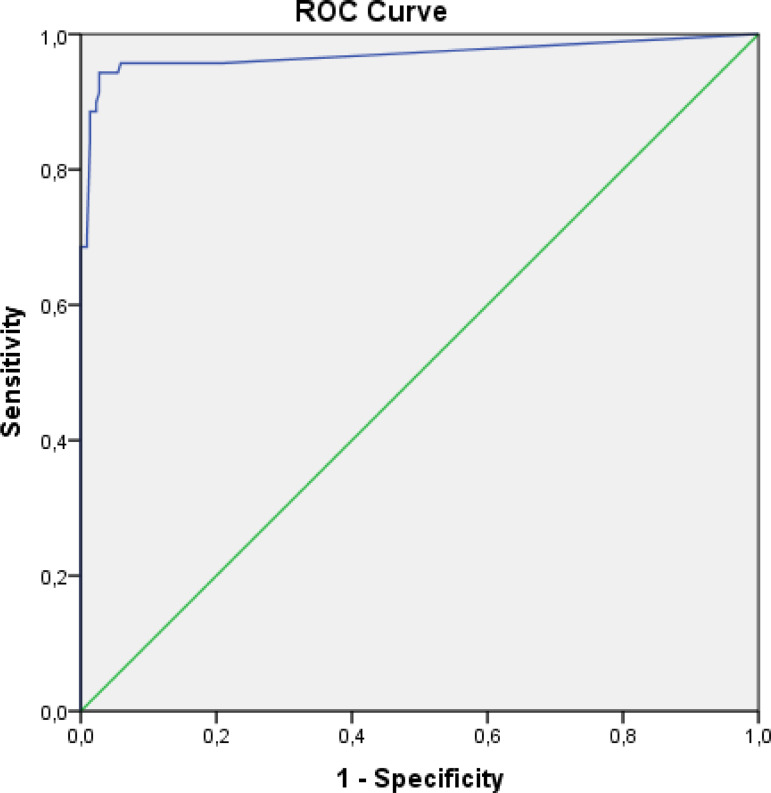
Receiver operating characteristics (ROC) curve for vasoactiveinotropic score — area under the ROC (95% confidence interval): 0.969 (0.938-1.000) (P<0.001).

Receiver operating characteristics (ROC) analyses were calculated to estimate combined morbidity and mortality development according to VIS value. An optimal cutoff point for VIS of 5.5 could predict combined morbidity and mortality as a reasonable sensitivity and maximal specificity - AuROC (95% CI): 0,969 (0,938-1,000) (P<0,001), sensitivity was 0,90 and specificity was 0,88 (Figure 1).

The average EuroSCORE and EF were 0.98 (0.5-6.75) and 55% (30-65), respectively. CPB time was 115.5 minutes (38-260), ACC time was 68 minutes (18-200), intubation duration was seven hours (1.7-89.5), and ICU length of stay was 47 hours (12.7-720). All patients underwent late extubation following CABG operation. The development of combined morbidity and mortality was significantly higher in patients with high EuroSCORE, low EF, long CPB and ACC times, and high VIS value (Table 1).

Combined morbidity and mortality rates of our patients were 23.8%, and the distribution of combined morbidity and mortality, according to VIS, is shown in Table 2.

**Table 2:** Distribution of combined morbidity and mortality according to VIS value.

	VIS < 5.5	VIS > 5.5	*P*-value*
Mortality			
Death	3	5	0.03
Morbidity			
Mechanic circulatory support	0	13	<0.001
Renal ınsufficiency	2	15	0.001
Cardiac arrest	2	8	0.002
Arrhythmia	5	8	0.02
Central nervous system damage	3	5	0.02
Combined morbidity and mortality	15	54	0.001

* Chi-square test

VIS=vasoactive-inotropic score

Low EF, prolonged operation time, high EuroSCORE, and high VIS are independent factors in the early postoperative period for the development of combined morbidity and mortality in patients with elective CABG operation (Table 3).

**Table 3:** Calculation of independent risk factors for development of combined morbidity and mortality.

	B*	SE	OR (95% CI)	*P*-value**
Age	0.012	0.033	1.012(0.949-1.080)	0.710
Sex	-0.085	0.648	0.919(0.258-3.274)	0.896
EF	-0.090	0.030	0.914(0.861-0.970)	0.003
CPB time	0.067	0.016	1.069(1.036-1.103)	<0.001
ACC time	-0.038	0.021	0.963(0.924-1.003)	0.072
EuroSCORE	1.407	0.445	4.083(1.707-9.764)	0.002
VIS	3.045	0.559	21.012(7.031-62.796)	<0.001

* B means ‘beta’ and it is the unstandardized regression weight

** Binary logistic regression analysis

ACC=aortic cross-clamp; CI=confidence interval; CPB=cardiopulmonary bypass; EF=ejection fraction; EuroSCORE=European System for Cardiac Operative Risk Evaluation; OR=odds ratio; SE=standard error; VIS=vasoactive-inotropic score

## DISCUSSION

We investigated the risk factors for early postoperative mortality and morbidity in CABG planned under elective conditions along with CPB. High VIS calculated at the end of the surgery, high EuroSCORE, low EF, and long CPB time were independent risk factors that increase mortality and morbidity in the early postoperative period.

Cardiac dysfunction, especially in the first 30 days postoperatively, is a significant complication that may cause high mortality and morbidity in patients with open-heart surgery^[10,11]^. Therefore, it is essential to predict poor postoperative outcomes in patients undergoing cardiac surgery^[10,11]^. Many clinical, laboratory, and scoring systems such as demographic factors, cardiac performance, planned surgical type and procedure, EuroSCORE, and VIS have been used to predict high morbidity and mortality in the early postoperative period^[4,5]^.

In the literature, many studies are investigating the relationship between high VIS and morbidity and mortality after open-heart surgery^[6,7,12,13]^. VIS was calculated at different times, and high VIS was a predictive value for high mortality and morbidity postoperatively^[6,7,12,13]^.

Inotropic use is frequently required in the cardiac surgery intraoperatively^[7,14,15]^. Besides, the inotropic drug requirement is influenced by many factors such as patient characteristics, surgical procedure, CPB and ACC times, amount of bleeding, and type of treatment given in the ICU^[7,14,15]^. We performed VIS measurement at the end of the surgical procedure, where the patient was most stable, and the factors affecting inotropic use were minimized. Also, another factor that determines the inotropic need is the type of surgical procedure to be performed on the patient. The patient population in our study was limited to CABG operation, unlike the literature. We think that the high VIS score is a good predictor of developing postoperative morbidity and mortality.

Studies are indicating that high EuroSCORE is a predictive value for postoperative mortality and morbidity in patients with open-heart surgery^[16-20]^. Similarly, we found that high EuroSCORE is an independent predictive value for early postoperative mortality and morbidity in open-heart surgery. While EuroSCORE is calculated preoperatively, VIS is calculated intraoperatively. However, inotropic drug is not always required in all patients who underwent open-heart surgery. Therefore, we think that the most objective evaluation of postoperative risk factors in open-heart operation should be performed with both EuroSCORE and VIS.

Long ACC and CPB times cause more myocardial dysfunction and a high inflammatory process^[21-24]^. Long CPB time may result in postoperative bleeding and morbidity caused by excessive blood and blood product transfusion^[21-24]^. In our study, long ACC and CPB times were risk factors affecting morbidity and mortality. However, CPB time alone was found to be a statistically independent risk factor.

We found that high VIS values, poor cardiac performance (EF), long CBP time, and high EuroSCORE were independent risk factors for determining morbidity and mortality. What we want to emphasize is not to limit the inotropic drug whenever it is required. We propose the hypothesis that increased VIS scores for reasons such as patient's characteristics or duration of surgery are associated with poor postoperative outcomes.

### Limitations

There are some significant limitations to this study. Different surgical teams performed the operations. The research was conducted in a single center, with a limited number of patients and operations. Although the number of patients is statistically reliable, we believe that multicenter studies with a more significant number of patients will provide better results.

## CONCLUSION

VIS is the most critical and EuroSCORE is the second most important scoring systems. They independently predict combined morbidity and mortality in undergoing elective coronary artery bypass surgery.

**Table:** 

Authors' roles & responsibilities	
PKB	Substantial contributions to the conception or design of the work; final approval of the version to be published
FG	Analysis of data for the work; final approval of the version to be published
EK	Interpretation of data for the work; final approval of the version to be published
MEG	Interpretation of data for the work; final approval of the version to be published
AE	Substantial contributions to the conception or design of the work; final approval of the version to be published
TO	Substantial contributions to the conception or design of the work; final approval of the version to be published
